# Ocrelizumab Extended Interval Dosing in Primary Progressive Multiple Sclerosis: An Italian Experience

**DOI:** 10.2174/1570159X22666231002142709

**Published:** 2023-10-02

**Authors:** Aurora Zanghì, Diana Ferraro, Graziella Callari, Paola Valentino, Franco Granella, Francesco Patti, Giacomo Lus, Simona Bonavita, Maria Claudia Moretti, Carlo Avolio, Emanuele D’Amico

**Affiliations:** 1 Department of Medical and Surgical Sciences, University of Foggia, Foggia, Italy;; 2 University of Modena and Reggio Emilia, Moderna, Emilia-Ronagna, Italy;; 3 IRCSS G.Giglio, Cefalù, Italy;; 4 Azienda Ospedaliera Universitaria “Mater Domini”, Catanzaro, Italy;; 5 Unit of Neurosciences, Department of Medicine and Surgery, University of Parma, Parma, Italy and Multiple Sclerosis Centre, Department of General Medicine, Parma University Hospital, Parma, Italy;; 6 Department “G.F. Ingrassia”, MS Center University of Catania, Catania, Italy;; 7 Multiple Sclerosis Center, II Division of Neurology, Department of Clinical and Experimental Medicine, Second University of Naples, Naples, Italy;; 8 Dipartimento di Scienze Mediche e Chirurgiche Avanzate, Università della Campania Luigi Vanvitelli, Piazza Miraglia, 2, 80138 Naples, Italy

**Keywords:** Ocrelizumab, primary progressive multiple sclerosis, extended interval dose, confirmed disability progression, anti CD20 therapies, COVID’19 pandemic

## Abstract

**Background:**

The intervals between two courses of anti CD20 therapies in the COVID19 pandemic era provided the opportunity to individually delay therapy, known as extended interval dosing (EID).

**Methods:**

We collect real-world data on patients with primary progressive MS (PPMS) treated with Ocrelizumab (OCR) during the COVID’19 pandemic. The observation period in which the standard interval dosing (SID) or EID occurred (always a maintenance cycle, 600 mg) was from January 2020 to June 2021. All patients had two infusions during the observation period. Our first aim was to compare confirmed disability progression (CDP) between SID and EID patients.

**Results:**

From a total cohort of 410 patients treated with OCR, 96 patients fulfilled the inclusion criteria. All patients received two infusions during the index window, 71 received only SID infusions whilst 25 received at least one EID infusion throughout the entire follow-up. During the entire available follow-up (median 10 months, IQR 7-11), CDP was recorded in 5 patients (3/71, 4.2% SID and 2/25, 8% EID, V-Cramer = 0.141, *p*-value = 0.167). EID regimen did not influence the risk of CDP during the investigated follow up.

**Conclusion:**

In our multicentre real-world cohort, the EID regimen in PPMS patients did not result in increased CDP during the available follow-up.

## 1 INTRODUCTION

The intervals between two courses of B-cell depletion therapies are long, as treatment efficacy is determined by prolonged immunosuppression, and it was suggested to assess peripheral B-cell reconstitution, providing the opportunity to individually delay therapy, known as extended interval dosing (EID) [1]. Recent studies have shown about 20% of patients treated with rituximab and even fewer (~5%) with ocrelizumab (OCR) began to repopulate by 6 months, and CD19+ repopulation took longer than one year [2, 3]. Anti-CD20 agents deplete memory B-cells, which can last for years after treatment, raising concerns for long-term immunological complications, such as an increased risk of malignancy or hypogammaglobulinemia, with the latter associated with higher infection rates and reduced vaccine efficacy [4, 5]. The personalized therapy with monoclonal antibodies in Multiple Sclerosis (MS) reality is evolving fast, but data on anti-CD20 tailored treatment in Progressive Primary MS (PPMS) is lacking [6-8].

During the COVID-19 pandemic, international MS experts advised neurologists to consider Extended interval dosing (EID) between OCR infusions [9-11].

However, an EID for OCR has not been licensed or characterized, even if some real-world studies investigated its efficacy and safety, and it has usually been defined as an extension dose of ≥ 4 weeks [12].

In this study, we collected real-world data on patients with PPMS treated with OCR during the COVID’19 pandemic, and we aimed to compare standard interval dosing (SID) with EID regarding confirmed disability progression (CDP).

## 2 METHODS

### 2.1 Setting

It is a multicentre real-world retrospective study from six tertiary Italian MS centers. The data entry portal was iMed^©^ portal. Anonymized clinical data of MS patients were extracted on October 31^st^, 2021.

### 2.2 Participants

We included all PPMS patients who had an indication of OCR in accordance with prescription laws and treatment procedures approved by European and Italian Medicines Agencies [13, 14].

Inclusion criteria were: 1) patients with PPMS diagnosis according to the revised McDonald criteria [15]; 2) to have already completed the first initial treatment cycles of OCR (2 × 300 mg with a 2-week interval). Patients without follow-up data were excluded.

### 2.3 Procedures and Covariate Definition

The observation period in which either the SID or EID took place (always related to the maintenance cycle, 600 mg) was from January 2020 to June 2021.

The SID was defined as a regular maintenance interval of OCR infusion every 6 months, whereas the EID group included patients with OCR infusion delay of at least 4 weeks (6 months + ≥ 4 weeks).

Three infusions were considered in defining SID *vs.* EID, defined as follows: the last pre-COVID19 infusion (second 300 mg cycle or 600 mg maintenance infusion) before January 2020 (infusion a), while infusions b and c (always 600 mg standard maintenance dose) were the subsequent infusions, administered between January 2020 and June 2021; infusion c was the last OCR infusion. According to this definition, we considered a single interval from infusions a to c, defined as the a-c interval.

Disability was assessed by Expanded Disability Status Scale (EDSS) by a Neurostatus-certified MS specialist [16]. Magnetic resonance imaging (MRI) data were acquired on 1.5-T scanners (the same at each center from baseline to the end of the follow-up) and included T2- and pre- and post-contrast T1-weighted sequences. Postcontrast T1-weighted sequences were acquired after intravenous injection of gadolinium contrast agent (0.1 mmol/kg). A cerebral MRI acquired within three months before the infusion was considered the baseline MRI. MRI and clinical visits were scheduled every six months.

Peripheral blood CD19+ B-cell counts before each infusion and depletion were defined as < 10 cells/μL. The vaccination status against the severe acute respiratory syndrome coronavirus 2 (SARS-CoV-2) was recorded [17] and reasons for infusions delays were also collected.

### 2.4 Study Outcomes

Our first aim was to compare CDP after the last OCR infusion between SID and EID regimens. CDP was determined by standardized neurologic examinations, typically conducted at six-month intervals.

CDP was considered clinically relevant if 2 independent clinical assessments 3 months apart indicated an increase in the EDSS as follows: 1.5 points (baseline EDSS 0.0), 1.0 point (baseline EDSS 1.0-5.5), and 0.5 point (baseline EDSS ≥ 5.5).

Additionally, clinical or radiological disease activity was recorded (clinical relapses or T1 gadolinium-enhanced brain lesions as well as new/newly enlarging T2 brain lesions.

Safety concerns, such as concomitant infections, were collected.

### 2.5 Statistical Analysis

Data were described according to the nature of the variables. The data distribution was verified with the Shapiro-Wilk test. Analysis of variance with Welch correction was chosen to compare quantitative variables considering the different sample sizes of the investigating groups. Chi-squared or Fisher's exact test was applied when necessary to compare categorical variables.

CDP and MRI activity was compared with association analysis, calculating the V-Cramer index.

Logit regression models were built for the two outcomes, “CDP” and “MRI activity”, during the investigated follow-up.

For each model, the following variables were inserted: EID status (0 = SID, 1 = EID, dichotomic), age (continuous variable), sex (0 = male, 1 = female, dichotomic), number of disease-modifying therapies (DMTs) before enrolment (continuous variable), time on OCR therapy (continuous variable, months), relapses in the year before enrolment (0 = no/1 = yes, dichotomic), MRI activity (0 = no/1 = yes, dichotomic) in the year before enrolment, EDSS before infusion a (continuous), CD19+B cell depletion rate before infusion c (0 = depletion, 1 = not depleted, dichotomic). For dichotomic variables, the last one was considered as a reference. A multivariable analysis was built for all the variables with a *p*-value < 0.10 in the univariate analysis. The results were presented as odd ratios and the corresponding 95% confidence interval (CI). Significance was settled to .05. All analyses were performed using SPSS V.21 statistical software.

### 2.6 Ethics Statement

The study received approval from the Ethics Committee of the University of Foggia, Italy. The study was conducted in accordance with the ethical principles of the Declaration of Helsinki.

## 3 RESULTS

### 3.1 Participants

**Fig. (1):**
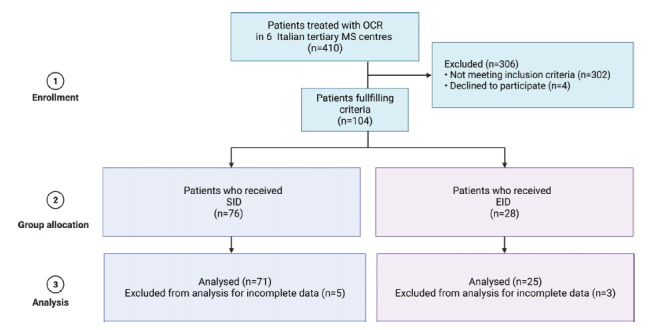
Flow chart of the study. **Abbreviations:** EID, extended interval dosing; MS, multiple sclerosis; OCR, ocrelizumab; SID, standard interval dosing. Created with Biorender.com.

From a total cohort of 410 MS patients treated with OCR, 96 PPMS patients fulfilled the inclusion criteria (Fig. **1**).

All patients received two infusions along a-c interval; 71 received only SID infusions, while 25 received at least one EID infusion. The mean EID duration during the a-c interval was 14.3 ± 3.4 months.

Table **1** shows the baseline characteristics of EID patients compared to SID ones. No differences were observed between the two groups.

The reason for the delay of the infusion was the same throughout the cohort, namely the patients' fear of accessing the hospital due to the risk of contracting the COVID-19 infection.

### 3.2 Follow Up Analysis

During the interval between the two infusions, no CDP or disease activity was recorded.

During the entire available follow-up (median 10 months, IQR 7-11), CDP was recorded in 5 patients (3/71, 4.2% SID and 2/25, 8% EID, V-Cramer = 0.141, *p*-value = 0.167.

One patient had a relapse (this patient had only SID infusions). Six patients (4/71, 5.6% SID and 2/25, 8% EID) had MRI activity with a median time of 3.9 ± 0.5 months from the last infusion.

In the univariable logit model for the outcome “CDP,” no variables were retained as predictors, so no multivariable models were built (Table **2**). In detail, being on SID/EID group did not influence the outcome (OR 3.09, 95% CI 0.58-16.43, *p* = 0.186).

In the univariable logit model for “MRI activity,” no variables were retained as predictors, so no multivariable models were built (Table **3**). In detail, being on SID/EID group did not influence the outcome (OR 0.68, 95% CI 0.11-4.00, *p* = 0.676).

### 3.3 Safety Concerns and Infection Risk

Generally, no serious infections were documented during the index window and the available follow-up.

Three patients got a SARS-CoV-2 infection during the index window; out of them, two were in the SID group and one was in the EID group. The mean time from the last OCR infusion was 2.3 ± 0.9 months. None had a serious infection, and none required hospitalization. No pneumonia was reported.

## 4 DISCUSSION

In our multicentre real-world cohort, the EID regimen in PPMS patients did not influence disease trajectory along the available follow-up.

Anti-CD20 personalized therapy is evolving fast, but the literature on B-cell-tailored personalized treatment in PPMS is lacking. Few recent studies suggested EID as a strategy to minimize adverse events while maintaining efficacy in the RRMS population, while another study reported an increased risk of MRI activity [1, 18-20].

Such data is of great importance, but all (mostly retrospective) cohorts have short durations of follow-up and lack control groups. Moreover, among the studies, the strategies of EID are different, some investigating a set interval extension and others only re-dosing based on the percentage or absolute number of CD19+ or CD27+ B-cells [19].

OCR represents the unique approved therapy for the management of PPMS, based on data from the ORATORIO clinical trial, but it is still challenging to determine which patients benefit most from OCR [21]. In such a population, further optimizing therapeutic MS healthcare regarding complications, costs and economic convenience represents a major goal [22].

Additionally, the COVID-19 pandemic has raised several safety concerns, especially for PPMS patients. It may be stated that cancellation of routine neurology examinations due to the fear of being infected with the virus and the socioeconomic effects of the epidemic are other factors contributing generally to a lower quality of life [23].

The main limitation of our study is represented by the selection biases of the target patients to whom this approach should be reserved that have certainly limited the robustness of the data on disease activity which, combined with the relatively short observation period and the small sample size, does not allow us to give an answer of certainty about the possibility of an EID in PPMS patients receiving OCR therapy.

It might be important to determine whether the infusion interval extension has a significant impact on disease progression over a longer period in big cohort future prospective studies [24, 25]. Additionally, further studies should also consider biomarkers of disease progression as neurofilament light chains to prognosticate disease course in such a minority of MS patients [26].

On the other hand, although no definitive conclusions could be applicable on this basis, we have reported data on a silent minority of MS patients (PP) during such a delicate period as the COVID19 pandemic in Italy, one of the first countries to face the emergency in the absence of data on MS DMTs.

In conclusion, although EID in PPMS is of interest and could decrease costs and safety concerns, we should be cautious of disability progression when considering infusion interval extension. We need randomized controlled trials to investigate anti-CD20 EID regimens in such a minority of the MS population.

## Figures and Tables

**Table 1:** Whole cohort and SID/EID groups characteristics.

Variables**	**TOTAL ** **(n = 96)**	**SID ** **(n = 71)**	**EID (n = 25)**	*P*-value*
Interval a-c duration (months)	21 ± 12.2	11.7 ± 1.9	14.3 ± 3.4	**0.003**
Age	52.7 ± 9.2	53.1 ± 9.3	51.4 ± 9.2	0.432
Female, n (%)	54(56.3)	42 (59.2)	12(48)	0.101
Disease Duration (years)	10.1 ± 8.5	10.5 ± 8.9	8.8 ± 6.9	0.337
Naive, n (%)	43 (44.8)	32 (45.1)	11 (44)	0.724
N. of DMTs before OCR	1.2 ± 1.4	1.3 ± 1.4	1.1 ± 1.3	0.663
EDSS before infusion a, median (IQR)	6.0 (4.0-6.5)	6.0 (4.0-6.0)	6.0 (4.0-6.5)	0.343
Patients relapsing in the year before infusion a, n (%)	12 (12.5)	9 (12.6)	3 (12)	0.942
Patients with MRI activity in the year before infusion a, n (%)	17 (17.7)	13 (18.3)	4 (16)	0.194
Time on OCR (from starting to infusion a, months)	30 ± 10.4	29.9 ± 10.6	32.9 ± 8.6	0.096
Vaccination completed against Sars-Cov2, n (%)	43(44.8)	32 (45.1)	11 (44)	0.558
CD19+ B cells depletion before Infusion a, n (%)	56(58.3)	43(60.6)	13(52)	0.487
CD19+ B cells depletion before Infusion b, n (%)	70(72.9)	50(70.4)	20(80)	0.439
CD19+ B cells depletion before Infusion c, n (%)	65(67.7)	49(69)	16(64)	0.630

**Note:** *via Anova with Welch correction and Chi-squared test/Fisher exact test; **data are reported as mean±standard deviation when otherwise specified.

**Abbreviations:** DMTs, disease modifying therapies; EID, extended interval dosing; EDSS, expanded disability status scale; MRI, magnetic resonance imaging; N. number; OCR, ocrelizumab; Sars-Cov2, severe acute respiratory syndrome coronavirus 2.

**Table 2:** Univariate model for “confirmed disability progression”.

Independent Variable*	**Univariable Analysis**	
	**OR (95% CI)**	***P*-value**
Sex	2.98 (0.48-9.24)	0.890
Age (years)	0.94 (0.86-1.04)	0.287
Disease duration (years)	0.96 (0.86-1.08)	0.582
N. of DMTs before OCR	1.06 (0.59-1.92)	0.832
EDSS before infusion a	0.91 (0.47-1.72)	0.786
Patients with relapses in the year before infusion a	0.55 (0.29-3.21)	0.618
Patients with MRI activity in the year before infusion a	1.63 (0.69-3.21)	0.128
Time on OCR therapy (from start to infusion a, months)	1.08 (0.98-1.19)	0.113
EID	3.09 (0.58-16.43)	0.186
CD19+ B-cell depletion ratebefore infusion c	2.50 (0.27-12.36)	0.412

**Abbreviations:** OR, odds ratio; DMTs, disease modifying therapies; EID, extended interval dosing; EDSS, expanded disability status scale; MRI, magnetic resonance imaging; N. number; OCR, ocrelizumab.

**Note:** *For dichotomic variables, the last category was used as reference.

**Table 3:** Univariate model for “MRI activity”.

Independent Variable*	**Univariable Analysis**	
	**OR (95% CI)**	***P* value**
Sex	1.80 (0.50-5.14)	0.789
Age (years)	1.05 (0.91-1.09)	0.935
Disease duration (years)	0.61 (0.35-1.04)	0.073
N. of DMTs before OCR	0.25 (0.04-1.42)	0.121
EDSS before infusion a	0.64 (0.40-1.04)	0.073
Patients with relapses in the year before infusion a	0.41 (0.08-3.12)	0.663
Patients with MRI activity in the year (1-3) before infusion a	1.55 (0.98-6.47)	0.291
Time on OCR therapy (from start to infusion a, months)	0.92 (0.84-1.01)	0.087
EID	0.68 (0.11-4.00)	0.676
CD19+ B-cell depletion rate before infusion c	1.05 (0.18-6.07)	0.955

**Abbreviations:** OR, odds ratio; DMTs, disease modifying therapies; EID, extended interval dosing; EDSS, expanded disability status scale; MRI, magnetic resonance imaging; N. number; OCR, ocrelizumab.

**Note:** *For dichotomic variables, the last category was used as reference.

## Data Availability

Anonymised data will be shared upon request from any qualified investigator for the sole purpose of replicating procedures and results presented in the report, provided that data transfer is in agreement with EU legislation on the general data protection regulation.

## LIST OF ABBREVIATIONS

EID: Extended Interval Dosing
OCR: Ocrelizumab
MS: Multiple Sclerosis
PPMS: Progressive Primary MS
SID: Standard Interval Dosing
CDP: Confirmed Disability Progression
SARS-CoV-2: Severe Acute Respiratory Syndrome Coronavirus 2

## STANDARDS OF REPORTING

STROBE checklist is available on the publisher’s website.
